# Complete Nucleotide Sequence of a South African Isolate of Grapevine Fanleaf Virus and Its Associated Satellite RNA

**DOI:** 10.3390/v5071815

**Published:** 2013-07-17

**Authors:** Renate L. Lamprecht, Monique Spaltman, Dirk Stephan, Thierry Wetzel, Johan T. Burger

**Affiliations:** 1 Department of Genetics, Stellenbosch University, Private Bag X1, Matieland 7602, South Africa; E-Mails: renatelamprecht@sun.ac.za (R.L.L.); 15660060@sun.ac.za (M.S.); dstephan@sun.ac.za (D.S.); 2 RLP Agroscience, AlPlanta–Institute for Plant Research, Breitenweg 71, Neustadt an der 67435 Weinstrasse, Germany; E-Mail: thierry.wetzel@agroscience.rlp.de

**Keywords:** Grapevine fanleaf virus, Arabis mosaic virus, satellite RNA, phylogenetics, full-length infectious cDNA clones, herbaceous hosts

## Abstract

The complete sequences of RNA1, RNA2 and satellite RNA have been determined for a South African isolate of Grapevine fanleaf virus (GFLV-SACH44). The two RNAs of GFLV-SACH44 are 7,341 nucleotides (nt) and 3,816 nt in length, respectively, and its satellite RNA (satRNA) is 1,104 nt in length, all excluding the poly(A) tail. Multiple sequence alignment of these sequences showed that GFLV-SACH44 RNA1 and RNA2 were the closest to the South African isolate, GFLV-SAPCS3 (98.2% and 98.6% nt identity, respectively), followed by the French isolate, GFLV-F13 (87.3% and 90.1% nt identity, respectively). Interestingly, the GFLV-SACH44 satRNA is more similar to three Arabis mosaic virus satRNAs (85%–87.4% nt identity) than to the satRNA of GFLV-F13 (81.8% nt identity) and was most distantly related to the satRNA of GFLV-R2 (71.0% nt identity). Full-length infectious clones of GFLV-SACH44 satRNA were constructed. The infectivity of the clones was tested with three nepovirus isolates, GFLV-NW, Arabis mosaic virus (ArMV)-NW and GFLV-SAPCS3. The clones were mechanically inoculated in *Chenopodium quinoa* and were infectious when co-inoculated with the two GFLV helper viruses, but not when co-inoculated with ArMV-NW.

Grapevine fanleaf virus (GFLV) is the causative agent of grapevine degeneration disease, and infected grapevines (*Vitis vinifera*) display symptoms that include degeneration and malformation of berries, leaves and canes [1]. The disease occurs worldwide where *V. vinifera* is cultivated and the vector is present, and it is considered the most important viral pathogen of grapevine in Europe. In South Africa, GFLV infections occur predominantly in the Breede River Valley in the Western Cape, due to the prevalence of its nematode vector, *Xiphinema index* [2]. GFLV is classified in the genus *Nepovirus*, family *Secoviridae*, with a genome that consists of two equally important positive sense, single-stranded RNA segments, RNA1 and RNA2 [3]. RNA1 (approximately 7.4 kb) encodes the proteins necessary for replication, while RNA2 (approximately 3.8 kb) encodes products that are involved in cell-to-cell movement and coating of the viral RNAs [4,5,6]. Both RNA1 and RNA2 are required for infection. GFLV and Arabis mosaic virus (ArMV) are serologically distant related viruses [7]. Some GFLV and ArMV isolates have been shown to support the replication of large satellite RNAs (satRNA) [8,9,10,11]. Satellite RNAs are dependent on the helper virus genome for replication, encapsidation and systemic spread [12]. Several satRNAs have been described for various ArMV isolates; the large satRNAs of isolates, ArMV-hop, ArMV-lilac, ArMV-p119, ArMV-NW, ArMV-P116 and ArMV-J86, are all between 1,092–1,139 nt in length [9,10]. The first GFLV-associated satRNA described was from the French isolate, F13. The GFLV-F13 satRNA is 1,114 nt in length and has no significant sequence homology to its helper virus, except for the first 10 nucleotides present in the 5' UTRs of both RNA1 and RNA2. The significance of this consensus sequence remains to be demonstrated [8,13]; however, it was suggested that the replication determinants are present in the 5' UTR and 5' end of the open reading frame (ORF) of GFLV satRNAs [11]. Recently, two new GFLV-associated satRNAs from California were completely sequenced. The satRNAs of GFLV-R2 and R6 were 1,140 nt in length and shared higher nucleotide identities to the ArMV-J86 and ArMV-NW large satRNAs than to the GFLV-F13 satRNA [11]. To date, the RNA1 and RNA2 of only four GFLV isolates (GFLV-F13, GFLV-WAPN173, GFLV-WAPN6132 and GFLV-SAPCS3) have been completely sequenced [14,15,16,17]. However, isolates GFLV-F13 and ArMV-NW are the only isolates that have their full genome, as well as their large satRNAs completely sequenced [10,13,14,15,18,19]. Here, we report the complete sequences of RNA1, RNA2 and the satRNA of a South African GFLV isolate, GFLV-SACH44. The GFLV-SACH44 satRNA is more closely related to three ArMV satRNAs than to the other GFLV-associated satRNAs sequenced to date. Full-length cDNA infectious clones of the GFLV-SACH44 satRNA were constructed and were able to replicate in herbaceous hosts when mechanically co-inoculated with the helper virus isolates, GFLV-NW [18] or GFLV-SAPCS3 [17], but not with ArMV-NW [18,19]. 

GFLV-SACH44 was sampled in 2010 from a grapevine plant (*Vitis vinifera* cv. Chardonnay) collected in the Robertson wine-growing region of South Africa. Total RNAs were extracted from grapevine leaves using a Cetyltrimethylammonium bromide CTAB method [20]. High fidelity enzymes for cDNA synthesis (Superscript III Reverse Transcriptase, Invitrogen) and PCR (Ex Taq DNA Polymerase, Takara) were used. Primers for cDNA synthesis and PCR of GFLV-SACH44 RNA1 and RNA2 were initially designed from the South African isolate, GFLV-SAPCS3 [17], and, subsequently, from newly generated GFLV-SACH44 sequences. Primers for cDNA and PCR for GFLV-SACH44 satRNA were designed from conserved areas obtained from alignments of GFLV-F13 satRNA (GenBank Accession no. NC003203) and ArMV-NW satRNA (Accession nos. DQ187317 and DQ187315) full-length nucleotide sequences. Refer to Supplementary Table 1 for primer details. The resulting PCR products were purified and cloned, and at least three clones from each of the PCR products was sequenced in both directions. The nucleotide sequence at the 5' ends of GFLV-SACH44 RNA1, RNA2 and the satRNA were determined using a 5'-RACE System for Rapid Amplification of cDNA Ends (Invitrogen) following the manufacturer’s instructions. Primer dT(17) [21] was used for cDNA synthesis for the determination of the 3' terminal sequences of GFLV-SACH44 RNA1, RNA2 and the satRNA. All the sequences generated from the overlapping amplicons were used to build contiguous sequences of both genomic and satRNAs using CLC Main Workbench version 6.5 (CLC Bio). The full-length nucleotide and amino acid sequences of GFLV-SACH44 RNA1, RNA2 and the satRNA were compared to the full-length sequences of other GFLV and ArMV isolates by performing multiple sequence alignments using ClustalW [22]. The nucleotide and protein sequence identities, pairwise distance calculations and phylogenetic analyses were performed using the MEGA 5 analysis package [23].

**Table 1:** Shared sequence identities (closest and most distant) of GFLV-SACH44 and other Grapevine fanleaf virus (GFLV) or Arabis mosaic virus (ArMV) full-length sequences. satRNA, satellite RNA.

GFLV-SACH44 Segment	Closest Nucleotide (nt) and Amino Acid (aa) Identities	Lowest Nucleotide and Amino Acid Identities
RNA1	GFLV-SAPCS3: 98.2% (nt) and 99% (aa) GFLV-F13:2 87.3% (nt) and 93.8% (aa)	GFLV-WAPN6132 86.1% (nt) and 91.8% (aa)
RNA2	GFLV-SAPCS3 98.6% (nt) and 99.1% (aa) GFLV-F13 90.1% (nt) and 96% (aa)	GFLV-Ghu 84.4% (nt) and 90.2% (aa)
SatRNA	ArMV-lilac 87.4% (nt) and ArMV-P119 80.3% (aa) ArMV-P119 87.2% (nt) and ArMV-P116 79.1% (aa)	GFLV-R2 71.0% (nt) and ArMV-hop 64.2% (aa)

For the construction of the GFLV-SACH44 satRNA full-length cDNA clone, the entire GFLV-SACH44 satRNA was amplified as one fragment with primers that added a 5' terminal *Asc*I and a 3' terminal *Bsp*120I restriction enzyme site to the PCR product. The entire satRNA fragment was cloned into a TA cloning vector (pGEM-T Easy, Promega), from which it was digested with *Asc*I and *Bsp*120I (Fermentas) and cloned into an expression vector L140 [24], a modified pBluescript II SKM (Stratagene) vector that contains a double enhanced Cauliflower mosaic virus (CaMV) 35S promoter [25] and a self-processing hammerhead ribozyme sequence [26]. To test the infectivity of the satRNA clones, two clones were selected (L140-GFLV-SACH44-satRNAfl constructs 3 and 12, 1 μg each) and individually mixed with 10 μL plant sap derived from ArMV-NW, GFLV-NW and GFLV-SAPCS3 infected *Chenopodium quinoa* leaves that were macerated in an inoculation buffer (30 mM K_2_HPO_4_, 50 mM glycine, 1% celite, 1% bentonite, pH 9.2). The DNA and plant sap mixture were mechanically rub-inoculated onto the top two leaves of *C. quinoa* plants (6–8 leaf stage). For controls, plants were mechanically inoculated with plant sap of either ArMV-NW, GFLV-NW or GFLV-SAPCS3 without plasmid DNA and healthy plant sap. GFLV or ArMV DAS-ELISA (Bioreba) was performed with the top systemic leaves of the inoculated *C. quinoa* plants ten days post-inoculation to confirm successful virus transmission. Total RNAs were extracted from the upper, newly expanded systemically infected leaves from DAS-ELISA positive plants. The presence of satRNA derived from L140-GFLV-SACH44-satRNAfl in the total RNA was tested by RT-PCR using gene-specific primers. To confirm that satRNA amplification from the systemic leaves was from the systemic spread of RNA and not from plasmid DNA contamination, the RT-PCR was repeated without reverse transcriptase. Furthermore, the infectivity of the L140-GFLV-satRNAfl clones (when co-inoculated with ArMV-NW and GFLV-NW) was screened by Northern blot analysis, as described by Sambrook *et al.* [27]. The probe for the Northern blot analysis was prepared by using 25 ng of a purified PCR product (a 300 bp PCR product from the coding region of GFLV-SACH44 satRNA) that was labelled with [α-32P]dCTP (3,000 Ci/mmol, Perkin-Elmer) using the DecaLabel DNA labelling kit (Fermentas). Fuji screens and a scan phosphorimager Pharaos FxPlus molecular imager (Bio-Rad) were used to visualize the hybridization signals.

The entire genome of the South African isolate, GFLV-SACH44, including its associated satRNA, was sequenced. The plant from which the GFLV-SACH44 was isolated was not infected with ArMV, as determined by DAS-ELISA (Bioreba). The two RNAs of GFLV-SACH44 were 7,341 nt and 3,816 nt in length, respectively, excluding the poly(A) tail. The 5' UTRs of GFLV-SACH44 RNA1 and RNA2 were 243 and 271 nt, respectively, and the 3' UTRs were 246 and 215 nt, respectively. The 5' UTR of GFLV-SACH44 had the same insertion, AA/GTCCGTT/CA, at position 73–98, also found in GFLV-SAPCS3, GFLV-Ghu [28] and other ArMV isolates, but not present in any other GFLV isolates sequenced to date. This suggests that GFLV-SACH44, like GFLV-SAPCS3, may have arisen from the same ancestor that may have originated from an interspecies recombination event in the 5' UTR region between GFLV-F13 type and ArMV-Ta type isolates [17]. One large open reading frame was predicted for both GFLV-SACH44 RNA1 and RNA2, encoding P1 (2,284 amino acids) and P2 (1,110 aa), respectively. The complete sequences of RNA1, RNA2 and the satRNA were deposited in the GenBank database with the accession numbers, KC900162 and KC900163, respectively. 

The entire GFLV-SACH44 satRNA was 1,104 nt in length, excluding the poly(A) tail. The GFLV-SACH44 satRNA had a 5' UTR of 14 nt, a 3' UTR of 74 nt in length and a predicted single ORF coding for P3 (338 aa). Nucleotide positions 1–17 were identical to the same region of GFLV-F13 satRNA and three ArMV satRNA isolates, while the first 11 nucleotides were identical to the same region of GFLV-R2 and GFLV-R6 satRNAs. Only one area in the GFLV-SACH44 satRNA fragment was found to be identical to the GFLV and ArMV genomes, and that was from nt positions 1–7. The complete sequences of the GFLV-SACH44 satRNA were deposited in the GenBank database with the accession number, KC900164.

To determine the pairwise distances of GFLV-SACH44 RNA1 and RNA2, multiple full-length nucleotide and amino acid sequence alignments were performed with other full-length GFLV isolates. Likewise, to determine the pairwise distances of the satRNAs, full-length nucleotide and amino acid sequence multiple alignments were performed with the satRNA of GFLV-SACH44 and other GFLV- and ArMV-satRNAs. The closest and most distant nucleotide and amino acid identities shared with GFLV-SACH44 RNA1, RNA2 and satRNA are listed in Table 1. Interestingly, the GFLV-SACH44 satRNA was more closely related to the ArMV-Lilac satRNA (87.4%) compared to the GFLV-F13 satRNA (81.8%), while it was most distantly related to the GFLV-R2 satRNA (71.0%) Nucleotide identities of other isolates are shown in the phylogenetic trees in Figure 1. 

**Figure 1:**
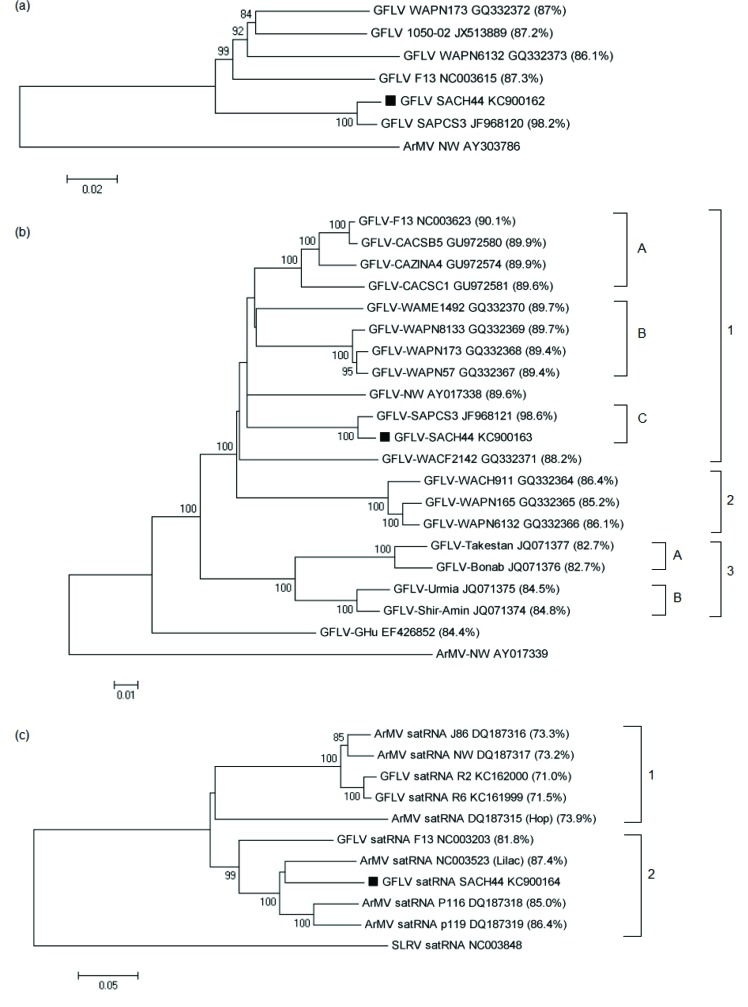
Phylogenetic trees based on full-length nucleotide sequences of (**a**) RNA1 of GFLV isolates; (**b**) RNA2 of GFLV isolates; and (**c**) satRNAs of GFLV and ArMV isolates. The nucleotide identities between GFLV-SACH44 and other GFLV or ArMV isolates are indicated in brackets. The accession numbers of the isolates are indicated next to the isolate names. GFLV-SACH44 is indicated on the tree as a solid block. For phylogenetic analysis of RNA1 and RNA2, ArMV-NW was used as an outgroup (Accession nos. AY303786 and AY017338, respectively), and strawberry latent ringspot virus (SLRSV) satRNA (Accession no. NC003848) was used as an outgroup for satRNA phylogenetic analysis. All the phylogenetic trees were constructed using the neighbor-joining method. The percentage of replicate trees in which the associated taxa clustered together in the bootstrap test (1,000 replicates) is shown next to the branches. Phylogenetic analysis was conducted in MEGA5 [23].

The phylogenetic tree based on full-length RNA1 sequences showed that GFLV-SACH44 and GFLV-SAPCS3 grouped together (Figure 1a), but were distinct from the other four full-length GFLV sequences. More GFLV RNA1 full-length sequences are, however, needed to clarify the phylogenetic relationship between GFLV isolates. A phylogenetic tree based on the full-length RNA2 sequences of GFLV-SACH44 and 19 other isolates revealed that all isolates, except for GFLV-Ghu, were grouped in three main clades (Figure 1b) that seem to be linked to geographic origin. Clade 1 mainly consists of Washington and Californian isolates and can be further divided into three sub-clades, of which GFLV-SACH44 and GFLV-SAPCS3 are placed separately in subgroup C. Clade 2 includes three Washington isolates, whereas clade 3 contains the Iranian isolates [29]. A phylogenetic tree based on the full-length sequences of GFLV and ArMV full-length satRNA sequences was constructed and revealed that there are two distinct clades (clade 1 and 2) (Figure 1c). Clade 1 included the GFLV-SACH44 satRNA, the lilac ArMV satRNA isolate, ArMV-P116 satRNA, ArMV-P119 satRNA and GFLV-F13 satRNA. The other clade, clade 2, included the satRNAs of the hop ArMV isolate, ArMV-J86, ArMV-NW and the recently described GFLV-R2 and GFLV-R6 satRNAs. The grouping of the two satRNA clades cannot be attributed to geographical origin, since the lilac ArMV satRNA isolate originated from the United Kingdom [9], and the other satRNAs in clade A were isolated from grapevines in different areas in Germany [10], France [8] and South Africa (this study). In clade 2, the satRNAs were isolated from hops in the United Kingdom [18], ArMV-J86, with unknown origin [10], ArMV-NW, from Germany [18], and the GFLV-satRNAs, from California [11]. 

Full-length cDNA clones of GFLV-SACH44 satellite RNAs constructed in this study were mechanically co-inoculated onto young *C. quinoa* plants, with plant sap derived from either GFLV-SAPCS3, GFLV-NW or ArMV-NW-infected *C. quinoa* (there was no satellite-free isolate GFLV-SACH44 available). The plants were tested by ELISA for the presence of the virus and by RT-PCR from total RNAs extracted from systemically infected leaves of ArMV or GFLV ELISA-positive plants for the presence of the satRNA. While the satRNA could be detected by RT-PCR and Northern hybridization in systemically infected leaves from plants inoculated with GFLV-SAPCS3 or GFLV-NW, it could not be detected in systemically infected leaves from plants inoculated with ArMV (not shown). No noticeable difference in symptoms was observed between GFLV-inoculated plants with or without the satellite. In Northern blot analysis (Figure 2), the clones produced a signal with the probe when it was co-infected with GFLV-NW, but not with ArMV-NW and, therefore, confirmed that the satRNA cDNA clones were infectious in *C. quinoa* with GFLV plant sap, but not with ArMV-NW.

The fact that the GFLV-SACH44 satRNA cDNA clones were replicated by two different GFLV isolates, but not with ArMV-NW plant sap, is interesting, considering that the phylogenetic analysis showed that the GFLV-SACH44 satellite was more closely related to other ArMV satellites than to GFLV satellites. This may indicate that these clones are infectious when co-inoculated with a homologous helper virus. However, a previous study reported that the GFLV-F13 satRNA cDNA clone was able to replicate when co-inoculated with an ArMV isolate (ArMV-S) as a helper virus [30]. Therefore, the recognition of the satellite RNA by the replication machinery is more complex than it appears, and additional sequences and infectious clones of satellites and their helper virus will be needed to address this question.

**Figure 2:**
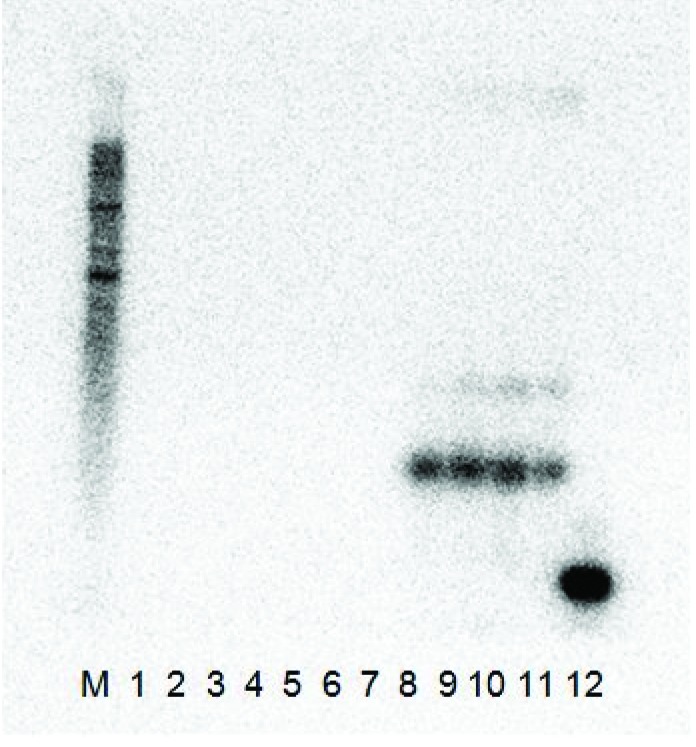
The Northern blot analysis results of the two L140-GFLV-satRNAfl clones 3 and 12 co-inoculated with ArMV-NW and GFLV-NW. Both constructs were shown to be infectious with only the GFLV-NW isolate as the helper virus and did not replicate with the ArMV-NW isolate as a helper virus. (**M**) RNA millennium (Ambion) marker, (**1**) ArMV-NW no satRNA control, (**2**–**3**) L140-GFLV-satRNAfl clone 3 co-inoculated with ArMV-NW, (**4**–**5**) L140-GFLV-satRNAfl clone 12 co-inoculated with ArMV-NW, (**6**) healthy *C. quinoa* control, (**7**) GFLV-NW no satRNA control, (**8**–**9**) L140-GFLV-satRNAfl clone 3 co-inoculated with GFLV-NW, (**10**–**11**) L140-GFLV-satRNAfl clone 12 co-inoculated with GFLV-NW and (**12**) positive PCR product control (probe ~ 400 bp).

## Supplementary Files

Supplementary File 1 Supplementary Table 1 (PDF, 160 KB)
