# Supplementary material for: Complete Nucleotide Sequence of a South African Isolate of Grapevine Fanleaf Virus and Its Associated Satellite RNA
Source: Viruses. 2013 Jul 17;5(7):1815–23. doi: 10.3390/v5071815 (PMC3738963; doi:10.3390/v5071815)
Supplement: Supplementary File 1 — Supplementary Table 1 (PDF, 160 KB) [file viruses-05-01815-s001.pdf]

Supplementary Table 1: Primers used in determining the full-length sequences of GFLV-SACH44 RNA1, RNA2 and satRNA. Isolates that were used to design the primers are also indicated.

Primer Name	Primer sequence	Designed from	Position
**RNA1**			
GFLV RNA1 RACE GSP1	TTCACACCTTTGCTTTAG	GFLV-SAPCS3	514–531
GFLV RNA1 RACE GSP2	CGCTATCCTGCTCTTCCTTA	GFLV-SAPCS3	383–402
GFLV RNA1 RACE GSP3	AGTTCCTTAGCCTCCGCATT	GFLV-SAPCS3	298–317
GFLV RNA1 pF2	TCCAGCGAAGAGTTTGAGAA	GFLV-SAPCS3	83–103
CH44 RNA1 seq 703 F	TAACACCAAGGGAAAAGTCGTC	GFLV-SACH44	777–798
GFLV1 2a 1908 F	GACACTTTTCTTCGCCAG	GFLV-SAPCS3	1,908–1,925
CH44 RNA1 2131 F	CTGGGAGCTTTTCATATTA	GFLV-SACH44	2,131–2,150
GFLV RNA1 pR2	AGTAAGTTCGCCTATCGCCC	GFLV-SAPCS3	2,195–2,214
CH44 RNA1 seq 2701 F	TGGAGTGGTTATGCCAGGCA	GFLV-SACH44	2,701–2,720
GFLV1 02b 2772 F	GCGAGTTCTATGATTGATG	GFLV-SAPCS3	2,773–2,791
GFLV1 03a 4027 F	GTGATGGAGTTAAGAAGCTA	GFLV-SAPCS3	4,028–4,047
CH44 RNA1 seq 4282 R	ACTTCTCATACTTAATGGGTTGCG	GFLV-SACH44	4,259–4,282
GFLV RNA1 p4 F	ATTCCTTGAAGATATTTC	GFLV-SAPCS3	4,817–4,836
CH44 RNA1 4912 R	CAACTTCATCAAGCAGTTTC	GFLV-SACH44	4,893–4,912
CH44 RNA1 block2 seq F	GTGCCAGGTAGGCATCAATC	GFLV-SACH44	5,355–5,374
GFLV1 04a 5457 F	GATGGCTTGATTACGGACC	GFLV-SAPCS3	5,458–5,476
CH44 RNA1 block3 seq F	CTGGTGCTTATAAAGAGTTG	GFLV-SACH44	6,128–6,147
CH44 RNA1 3'UTR 6982 F	TGTGCAGGGGTCGCAACTAA	GFLV-SACH44	6,982–7,001
**RNA2**			
GFLV RNA2 RACE GSP1	AAGAAAACATCCGAACAG	GFLV-SAPCS3	439–456
GFLV RNA2 RACE GSP2	ACAGTTTGGCGGAAGGAGGA	GFLV-SAPCS3	381–400
GFLV RNA2 RACE GSP3	AGACAAATAAGACGCCCGCT	GFLV-SAPCS3	309–327
CH44 RNA2 134 F	CCAAAGCGAAGAGTTTAAGA	GFLV-SACH44	134–153
GFLV RNA1 pF2	TCCAGCGAAGAGTTTGAGAA	GFLV-SAPCS3	626–645
CH44 RNA2 686 R	CACAGTGGCCCGTATAAACC	GFLV-SACH44	667–685
CH44 RNA2 seq gap 3	TGAGCAAGGCCTACCGCT	GFLV-SACH44	1,270–1,287
CH44 RNA2 block3 seq F	GAGGCTGAACCCAGATTGAG	GFLV-SACH44	2,057–2,076
GFLV RNA2 pF3	CTGAACATACGTGATATGAT	GFLV-SAPCS3	2,156–2,175
GFLV RNA1 pR2	AGTAAGTTCGCCTATCGCCC	GFLV-SAPCS3	2,903–2,922
GFLV2 3743R	ACAACACACTGTCGCCACTAAAAGC	GFLV-SAPCS3	3,718–3,742
CH44 RNA2 3'UTR 3557 F	GCCTCGTCCAGGTTTCAGTT	GFLV-SACH44	3,556–3,575
**SatRNA**			
GFLV satRNA RACE GSP4	CTTTTCAGCAGGAGCCCAGA	GFLV-SACH44	813–832
GFLV satRNA RACE GSP5	AGATAGAAGTGAGGGTGAAA	GFLV-SACH44	538–557
GFLV satRNA RACE GSP6	CTGCTGTTTGTGTCCCTTCG	GFLV-SACH44	252–271
GFLV satRNA 174F	GTAAGCAAACGGACCT	GFLV and ArMV	173–189
GFLV satRNA dgR	ACCTTACGCAACATCCG	GFLV and ArMV	905–921
GFLV satRNA dgF	TTCACCCTCACTGCTATC	GFLV and ArMV	539–556
GFLV ic satRNA 1F Asc**I**	AA**GGCGCGCC**ATGAAAAATTTCTAT GGGTTCTCGT	GFLV-SACH44	1–25
GFLV ic satRNA pR2 Bsp120**I**	AA**GGGCCC**(Tx30)GAGTTGGCTAAT GAGCAACC	GFLV-SACH44	1,085–1,104
